# Time to Next Treatment as a Meaningful Endpoint for Trials of Primary Cutaneous Lymphoma

**DOI:** 10.3390/cancers12082311

**Published:** 2020-08-17

**Authors:** Belinda A. Campbell, Julia J. Scarisbrick, Youn H. Kim, Ryan A. Wilcox, Christopher McCormack, H. Miles Prince

**Affiliations:** 1 Department of Radiation Oncology, Peter MacCallum Cancer Centre, Melbourne, VIC 3000, Australia; 2 Department of Clinical Pathology, The University of Melbourne, Parkville, VIC 3010, Australia; 3 Department of Dermatology, University Hospital Birmingham, Birmingham B15 2TH, UK; jscarisbrick@nhs.net; 4 Department of Dermatology, Stanford Cancer Institute, Stanford, CA 94305, USA; younkim@stanford.edu; 5 Division of Hematology and Medical Oncology, Department of Internal Medicine, University of Michigan Rogel Cancer Center, Ann Arbor, MI 48109, USA; rywilcox@med.umich.edu; 6 Department of Surgical Oncology, Peter MacCallum Cancer Centre, Melbourne, VIC 3000, Australia; chris.mccormack@petermac.org; 7 Department of Dermatology, St Vincent’s Hospital Melbourne, Fitzroy, VIC 3065, Australia; 8 Department of Clinical Haematology, Peter MacCallum Cancer Centre and Royal Melbourne Hospital, Melbourne, VIC 3000, Australia; miles.prince@petermac.org; 9 Sir Peter MacCallum Department of Oncology, The University of Melbourne, Parkville, VIC 3010, Australia

**Keywords:** time to next treatment, primary cutaneous lymphomas, clinical trials, clinical endpoint, study design

## Abstract

Time to next treatment (TTNT) is an emerging endpoint in clinical studies of primary cutaneous T-cell lymphomas (CTCL), with utility as a surrogate marker for the “duration of clinical benefit”. TTNT provides a highly clinically meaningful endpoint that uniquely reflects not only the duration of treatment efficacy on disease and symptom control, but also incorporates the patient experience by accounting for patient compliance and tolerance to the studied therapy(s). Given the distinct challenges of pin-pointing the exact date of progression in patients with multi-compartmental CTCL, TTNT overcomes many of the shortcomings of conventional, disease-focused, clinical endpoints in primary CTCL research. Although widely accepted in clinical research for numerous other incurable malignancies, TTNT currently lacks a standardised definition. In this paper, we describe the value of TTNT as a clinical endpoint, review the applications of TTNT in primary CTCL research, and propose a standardised definition of TTNT to be applied in future clinical research of primary CTCL therapies.

## 1. Introduction

Patients with primary cutaneous T-cell lymphomas (CTCL) commonly endure high symptom burden over a lengthy clinical course, with available treatments frequently resulting in incomplete responses and risks of toxicities. As such, the durability of clinical benefit is an important consideration in the global assessment of the therapeutic impact of treatment options. To date, standardised measurement of the duration of therapeutic benefit, with applicability in both prospective clinical trials and retrospective evaluation of data sets, has been challenging. In 2011, standardised definitions to measure treatment response in CTCL were published by an international consortium [1], with the modified Severity Weighted Assessment Tool (mSWAT) recommended in the assessment of skin-response according to defined response criteria (Table 1). However, measurement of the durability of this response has been more problematic. For these multi-compartmental diseases, numerous variables cloud the reliability of conventional measures of the progression-free interval and durability of response, which rely on accurately pinpointing the date of disease progression or relapse according to tumour burden. As a surrogate for duration of clinical benefit (DOCB), time to next treatment (TTNT) is an emerging endpoint in the published literature of CTCL, and has been shown to be a highly clinically relevant endpoint in low grade, incurable malignancies. TTNT measures the interval from the date of initiation of a treatment to the date of commencement of the next line of therapy, thereby allowing for easy measurement of the period of therapeutic benefit. Furthermore, TTNT offers a better reflection of the patients’ treatment experiences than conventional disease-related endpoints by incorporating the time course of treatment tolerability and patient compliance. In clinical studies of CTCL, TTNT is used as an addition to conventional evaluation criteria and also as a stand-alone assessment of duration of treatment efficacy [2,3,4,5,6,7,8], but to date no standardised definition has been agreed. Herein, we review the use of TTNT in the published CTCL literature, and propose a new standardised definition that accounts for the unique characteristics and challenges of CTCL.

## 2. Limitations of Conventional Endpoints in Measuring the Durability of Treatment Response in CTCL

Measurement of the progression-free interval is a standard endpoint in oncology studies, with progression-free survival (PFS) and freedom-from-progression (FFP) commonly reported in the literature. Similarly, measurement of the duration of response (DOR) is also widely reported, representing the time from the date of achieving best response to the date of disease progression. However, unique to CTCL, there are several limitations that reduce the usefulness of these endpoints. The primary limitation of PFS, FFP, and DOR relates to the difficulty in pinpointing the date of disease progression in patients with CTCL. Firstly, disease progression may be mistakenly “over-called” by intermittent, reversible, and/or self-limiting “disease flares” secondary to exogenous causes, for example, infection-induced exacerbation of skin disease which responds to antibiotic therapy and improved skin care, or treatment-induced disease flare on initiation of immunomodulatory agents (particularly checkpoint inhibitors). Secondly, symptomatic worsening of disease (for example, pruritis) may occur without meeting the strict definition for disease progression. Thirdly, discordant disease responses in skin, nodes, viscera, or blood, can confuse the overall impression of DOR, triggering a disease progression event despite sustained response elsewhere. Fourthly, high-grade transformation may occur without meeting disease progression criteria according to disease bulk. And, finally, treatment-related toxicities may mimic the appearance of disease progression, including diffuse radiotherapy-induced skin reaction secondary to total skin electron therapy (TSE), cutaneous graft-versus-host disease following allogeneic transplantation, and mogamulizumab-associated rash mimicking the clinical and/or pathological appearances of CTCL.

Objectively defining the more subjective “duration of therapeutic benefit”, or DOCB, is also challenging in CTCL. This is particularly problematic in patients with discordant treatment responses, for example, sustained resolution of pruritic stage T1/2/4 skin-disease but with development of small T3 tumour(s) would trigger a “disease progression event” despite the patient continuing to experience subjective therapeutic and symptomatic benefits. Defining the DOCB is further complicated in the context of therapies requiring long-term maintenance doses and their associated secondary toxicities, thus impacting on the therapeutic ratio and quality of life benefits for these patients with CTCL. Additionally, measuring DOCB during long-term maintenance treatments may be affected by patient compliance, for example, patients finding the frequent ultraviolet light (UV) sessions too inconvenient may choose to discontinue treatment despite an objective cutaneous benefit. Further, compliance to patient-driven, supportive care regimens may also affect durability of symptom control, thus influencing the DOCB. And, finally, although health-related quality of life tools provide validated assessments of patient well-being and pruritus control [1], they are limited to prospective studies. Moreover, worsening pruritis resulting in additional anti-tumour therapy is not incorporated into standard progression criteria.

Definitions relating to the measurement of the durability of the therapeutic benefit have varied in the literature [9,10]. In 2013, a phase 2 trial of romidepsin for CTCL used “duration of clinically meaningful reduction in pruritus” [11]. This endpoint utilised a patient-assessed visual analogue scale: although useful in prospective study design, it is inherently problematic in retrospective studies. In 2015, DOCB was explored as a new endpoint in a pooled analysis of three prospective trials of low-dose TSE, using the definition of “time from initial response until the initiation of any total skin equivalent treatment (topical treatment to >50% of body surface, phototherapy, second TSE), systemic therapy, or progressive disease” [9]. This definition of DOCB has since proven useful in the context of subsequent prospective clinical trials [10], but is difficult to apply to retrospective studies unless the dates of initial response are rigorously defined and prospectively collected in patients’ records. Thus, the need was recognised for a surrogate endpoint for DOCB that is easily and objectively measurable, with applicability to both prospective and retrospective study designs.

## 3. TTNT in CTCL

Over the past 5 years, TTNT has been utilised and endorsed as a surrogate marker for DOCB in clinical studies of CTCL [2,3,4,5,6,7,8]. TTNT represents the interval from commencement of one treatment to initiation of the next line of therapy. Thus, TTNT incorporates the period of treatment delivery, the times taken to achieve initial and best responses, plus the durations of disease and/or symptom control. Uniquely, TTNT also offers clinical relevance beyond that of conventional measures of treatment efficacy by accommodating the patient experience in terms of treatment tolerability and patient compliance. For example, the progression-free interval offers an objective measure of disease control, but the reliability may be affected by premature discontinuation of therapy prior to disease progression, early cessation of maintenance therapy due to poor patient compliance, or patients electing to switch therapies due to poor treatment tolerance. Additionally, disease progression in one compartment (for example, the skin) may be addressed with localised therapy(s) without necessitating changes in systemic therapy if disease control is sustained in other compartment(s) (blood, nodes, and viscera). In this setting, TTNT offers superior measure of the durability of therapeutic benefit than measurement of the progression-free interval alone. Further, the easy measurability of TTNT overcomes the challenges imposed by conventional endpoints to pin-point dates of treatment response and disease progression. Importantly, TTNT offers clinical relevance to patients and physicians, beyond that of other markers of treatment efficacy.

## 4. TTNT in Published Retrospective Studies in CTCL

Recent publications have clearly demonstrated the applicability of TTNT as a tool to measure and compare the DOCB of multiple therapeutic agents in patients with CTCL. In two retrospective studies, TTNT was used as the primary endpoint to compare the efficacy of different treatments across single institutional databases [2,3]. These studies examined the TTNT of currently available “systemic” therapies, including psoralen plus UVA radiation (PUVA), chemotherapy, low dose methotrexate, α-interferon, histone deacetylase inhibitors (HDACi), oral retinoids (including bexarotene), the monoclonal antibody alemtuzumab, the fusion toxin denileukin difitox, high dose chemotherapy with autologous haematopoietic stem cell transplant, allogeneic haematopoietic stem cell transplant, extracorporeal photopheresis (ECP), and TSE. In both studies, chemotherapy had the shortest TTNT of all therapies, confirming the poor efficacy of this treatment in CTCL and prompting the authors to caution against the routine use of chemotherapy for CTCL [2,3]. As reported in the study from the Peter MacCallum Cancer Centre, the median TTNT of all treatments was 5.4 months (95% CI: 5.1–6.1), with the median TTNT of chemotherapy measuring 3.9 months (95% CI: 3.2–5.1), and only 10.7% of patients were free from further treatment at one year [2]. Similarly, in the study from the University of Michigan Comprehensive Cancer Centre, the TTNT following chemotherapy was also short with the mean TTNT measuring 5.1 months and only 7.5% of patients remaining free from next treatment at one year [3]. Additionally, the investigators reported longer TTNT associated with biological response modifiers, with α-interferon demonstrating improved efficacy in both early and advanced stages of disease in both studies [2,3]. This introduction of TTNT into CTCL research was ground-breaking: overcoming the limitations of conventional endpoints and allowing tangible measurement of treatment efficacy in the retrospective setting. Given the vast differences in toxicity profiles and modes of administration, TTNT was also valuable as an endpoint that considered tolerability and patient compliance. In these analyses, the strength of TTNT rested on the stability of the treating multi-disciplinary teams with consistent prescribing patterns. The authors of both studies acknowledged the clinically meaningful value of TTNT, combining disease progression, symptom control, and treatment tolerability into one endpoint [2,3].

Secondly, TTNT has been used to evaluate the DOCB of different therapeutic agents within the same pharmacological family. In this setting, prospective randomised studies are unlikely due to the rarity of CTCL and the requirement for industry cooperation. However, TTNT provides a clinically meaningful and tangible endpoint in the retrospective setting. A recent retrospective study of three HDACi (infusional romidepsin, oral vorinostat, and oral panobinostat) in patients with multiply-relapsed MF/SS, reported an overall median TTNT of 5.5 months (range, 1–124) with no significant differences found in TTNT between the three agents [4]. This study demonstrated the usefulness of TTNT as a surrogate endpoint for DOCB: TTNT considered not only the duration of disease control from the HDACi but also the patient experience, incorporating differences in patient compliance and tolerability which may have arisen from differing modes of administration and toxicity profiles.

Thirdly, TTNT has been applied towards individualising care for CTCL patients. Conventional-dose TSE is a well-established treatment for CTCL, and as a stand-alone therapy, it uniquely offers patients hope for a durable treatment-free period. However, in a retrospective study, TTNT was used to identify patient subgroups less likely to benefit from conventional-dose TSE [5]. In this study, SS patients derived very short palliative benefit from TSE monotherapy with median TTNT only 3.7 months (95% CI: 2.3–4.4) versus 10.9 months (95% CI: 5.1–20.3) for MF patients (HR = 4.5, 95% CI: 2.2–9.2, *p* < 0.001). In the patients with MF, those with heavily pre-treated disease derived significantly shorter TTNT than those receiving TSE earlier in their treatment journeys: median TTNT was 23.2 months (95% CI: 12.7–34.8) for patients with 0–2 prior lines of therapy, versus 7.1 months (95% CI: 3.4–10.9) for patients with ≥3 previous treatments (HR per additional prior treatment line = 1.13, 95% CI: 1.01–1.27, *p* = 0.031). The authors concluded that for optimal care of MF patients, earlier delivery of TSE in the treatment paradigm should be considered to maximise the DOCB and treatment-free interval [5]. Looking forward, we anticipate that TTNT will serve as a useful endpoint in future clinical studies to individualise patient care and optimise treatment sequencing for patients with CTCL.

Fourthly, TTNT has been used to assess the efficacy of more novel treatment regimens in patients with CTCL, and is now a recognised endpoint by regulatory authorities. Although prospective randomised studies remain the gold standard, TTNT in the retrospective setting has increasingly recognised value as a combined measure of disease and/or symptom control, tolerability, and patient compliance. A retrospective analysis of a new ECP regimen reported an impressive DOCB from ECP monotherapy in patients with erythrodermic MF/SS with median TTNT of 14 months, which was significantly longer than that of all other available systemic therapies including interferon-a (median TTNT 8 months, *p* = 0.0067), HDACi (median TTNT 7.5 months, *p* = 0.0003), novel immunotherapy agents (median TTNT 6.5 months, *p* = 0.028), low-dose methotrexate (median TTNT 2.5 months, *p* < 0.0001), and chemotherapy (median TTNT 3.0 months, *p* < 0.0001) [6]. Through the use of TTNT, this study provided unprecedented demonstration of the DOCB in patients with erythrodermic MF/SS, allowing recognition of ECP by regulatory bodies [12,13] and incorporation into accepted standard of care practices in Australia.

## 5. TTNT in Published Prospective Clinical Studies in CTCL

For the first time, TTNT has been recognised as a valid endpoint in prospective, randomised studies of CTCL [7,8]. These two international prospective trials have demonstrated the usefulness of TTNT to provide clinically meaningful assessment of treatment efficacy, considering differences in the toxicity profiles and modes of delivery, beyond the traditional markers of disease control. Further, these international, collaborative studies are the first to demonstrate the value of TTNT in the context of multi-centre trials.

In the first phase 3 study (ALCANZA), 128 patients with previously-treated CD30-positive CTCL were randomised to intravenous brentuximab vedotin or physician’s choice (oral methotrexate or oral bexarotene) [14]. The primary endpoint was objective global response lasting at least 4 months (ORR4) [14], with superior ORR4 in favour of brentuximab vedotin over physician’s choice: 54.7% versus 12.5% (*p* < 0.001) [15]. Given the differences in mode of administration and toxicity profiles, TTNT was also investigated as a clinically meaningful surrogate for DOCB that incorporates the patient experience [8,15]. In this context, TTNT provided a valuable and complementary endpoint to conventional measures. Defining TTNT as the time from randomisation to the date of first documentation of antineoplastic therapy or last contact date for those who did not receive further antineoplastic therapy, Ref. [8] the authors confirmed brentuximab vedotin to be associated with more durable clinical benefit, with median TTNT measuring 14.2 months versus 5.6 months for physician’s choice (HR = 0.269; 95% CI: 0.171–0.424; *p* < 0.001) [15].

In the second international phase 3 study (MAVORIC), 372 MF/SS patients were randomised to intravenous mogamulizumab or oral vorinostat, with crossover in the latter group upon disease progression or treatment-intolerance [7,16]. PFS was the primary endpoint, with superior median PFS seen in the group receiving mogamulizumab, 7.7 months (95% CI: 5.7–10.3) versus 3.1 months (95% CI: 2.9–4.1) in the vorinostat group (HR = 0.53; 95% CI: 0.41–0.69; *p* < 0.0001) [16]. Given the crossover design, the DOCB was an additionally important question, with post-hoc analysis confirming longer TTNT following mogamulizumab in both initial and crossover settings [7]. In this analysis, TNTT was defined as the time to the next line of significant therapy (systemic treatment, TSE, or PUVA): median TTNT was 11.0 months (95% CI: 8.8–12.6) in the group receiving mogamulizumab versus 3.5 months (95% CI: 3.1–4.3) in the group receiving vorinostat (*p* < 0.0001), with median TTNT of 10.1 months (95% CI: 8.0–12.6) in patients who crossed over to mogamulizumab [7]. Prolonged TTNT was also observed across stage grouping and disease type for patients receiving mogamulizumab [7,17]. The authors state that TTNT “represents an additional measure of clinical benefit and disease control in patients who may have progressed based on strict protocol definitions of progression” [7].

## 6. Use of TTNT in Other Diseases

TTNT is not unique to CTCL for the measurement of DOCB. TTNT is a well-accepted endpoint in clinical trials of patients with multiple myeloma [18,19,20,21,22,23,24,25,26,27,28]. It also features in the published literature of clinical studies of patients with chronic lymphocytic leukaemia [29,30,31,32,33,34], follicular lymphoma [35,36,37,38,39], mantle cell lymphoma [40], amyloidosis [41], Waldenstrom macroglobulinaemia [42,43], autoimmune haemolytic anaemia [44], metastatic melanoma [45], and metastatic breast cancer [46]. TTNT has also been used as a valid endpoint within cost-benefit analyses in several published health-economic analyses [19,24,27,29]. Interestingly, the use of TTNT has also been applied to the use of positron emission tomography for the purposes of prognostication [23,39]. Common to all are the underlying disease behaviours: typically demonstrating intermediate to long natural histories, accumulated exposures to multiple lines of therapy, and low likelihoods of cure. Collectively, these studies confirm the usefulness of TTNT where DOCB is the highest priority for optimal patient care.

## 7. Weaknesses of TTNT

We acknowledge that “no endpoint is perfect”. Perceived weaknesses of TNTT are the potential influences of the prescribing patterns of individual physicians, especially when selecting the next line of treatment and the timing of switching over. Differences in multidisciplinary care may also exist between centres and across geographic regions: the availability of treatments (for example, TSE, ECP, or new therapeutic agents) may drive treatment decisions and lead to evolving prescription patterns over time. Therefore, when TTNT was first adopted into clinical studies, it was confined to single institutional studies in the context of stable multidisciplinary teams [5]. However, the recent application of TTNT into international, collaborative studies has demonstrated that TTNT also has value as a clinical endpoint in the context of multi-centre trials [7,8].

Arguably, the strongest application for TTNT is in studies of advanced-stage disease where systemic therapy is the mainstay. Conversely, TTNT may not be suitable for studies of localised, skin-directed therapies (topical steroids, nbUVB/PUVA, localised radiotherapy) for early-stage disease or focal therapies where local symptom-control is the primary objective. Indeed, more specific definitions of TTNT could be considered depending on the context of the trial, for example “time to next whole skin treatment” in stage I CTCL, or “time to next systemic treatment” in advanced-stage CTCL. However, as a global measure of DOCB with application to both systemic and non-systemic therapies (for example, TSE, topical chemotherapy, future topical biological agents), we prefer to maintain the existing, well-utilized acronym, TTNT.

One other possible weakness may arise from subtle variations in TTNT definitions between studies. For example, in the multiple myeloma literature, TTNT has been variably defined including one study which defined TTNT as “the addition of a new agent > 60 days from index or as treatment restart following a >90-day therapy gap” [20]. This weakness is not unique to TTNT: consistent criteria and rules for analysis are necessary elements for all response criteria and endpoints. To overcome this limitation, we propose a multi-disciplinary, standardised definition of TTNT for future clinical trials in CTCL (Table 2).

## 8. Conclusions

TTNT is an emerging endpoint in clinical studies of CTCL. For CTCL patients with intermediate-long clinical courses, multiple therapeutic options, and no clearly defined optimal treatment sequence, TTNT represents a valuable tool to measure and compare the efficacy and durability of therapeutic benefit of established and novel therapies. TTNT is a unique and clinically meaningful endpoint, reflecting durations of disease and symptom control whilst also considering treatment tolerability and patient compliance. Furthermore, TTNT serves as an objective and tangible surrogate for DOCB, with established value in both retrospective and prospective study designs. We anticipate and encourage the increasing inclusion of this useful endpoint into clinical CTCL trial design, and propose standardisation of the TTNT definition accounting for the unique challenges faced in CTCL research and patient care.

## Figures and Tables

**Table 1:** Standardised skin response definitions (utilising the modified Severity Weighted Assessment Tool score) [1].

Response	Definition
Complete response	100% clearance of skin lesions.
Partial response	50–99% clearance of skin disease from baseline without new tumours (T_3_) in patients with T_1_, T_2_, or T_4_ only skin disease.
Stable disease	<25% increase to <50% clearance in skin disease from baseline without new tumours (T_3_) in patients with T_1_, T_2_, or T_4_ only skin disease.
Progressive disease	≥25% increase in skin disease from baseline, or New tumours (T_3_) in patients with T_1_, T_2_, or T_4_ only skin disease, or Loss of response: in those with complete or partial response, increase of skin score of greater than the sum of nadir plus 50% baseline score.
Relapse	Any disease recurrence in those with complete response.

**Table 2:** Proposed standardisation of the definition of time to next treatment (TTNT) for use in future clinical studies of primary cutaneous T-cell lymphomas (CTCL).

	Defined Parameters
Measurement	TTNT is measured from the date of initiation (first dose) of treatment, to the date of initiation of the next line of therapy.
Exclusions	“Next line of therapy” excludes skin-directed therapies including topical steroids, nbUVB/PUVA, or focal radiation where less than 50% of the skin area is irradiated. Note: the cut-off of 50% is arbitrary but consistent with prior definitions of DOCB [9,10].
No subsequent lines of anti-CTCL therapy	For patients who are not fit for active management (due to co-morbidities and/or poor performance state), or those who decline further anti-CTCL treatment, the “next line of therapy” is recorded from the date of commencement of “end-of-life care” [47] and/or the withdrawal of all anti-CTCL therapies.
Censoring	Patients who have not progressed to a subsequent line of therapy, will be censored at the date of last follow up or at death.
Short-term treatment gaps	Short-term treatment gaps within the one course of prescribed therapy do not trigger a “next line of therapy”, provided no progression of disease occurs during the during treatment break. Short-term treatment gaps are defined as treatment withheld for duration < 2 months, for the following reasons: intercurrent illness, lack of treatment availability, for relief of toxicities, or patient preference. For treatment gaps ≥ 2 months, re-commencement of therapy will constitute a subsequent line of therapy, thus triggering an event in the TTNT and re-starting the “TTNT clock”.
Maintenance therapies	Commencement of pre-planned consolidation/maintenance anti-CTCL therapy in a patient with controlled disease will not trigger a TTNT event.
Allogeneic transplantation	For patients undergoing allogeneic transplantation, TTNT is triggered at the date of commencement of the conditioning therapy. If TSE is incorporated pre-transplant, then the date of commencement of TSE should be the time point used to trigger the TTNT.
Prospective clinical trials	In the context of prospective clinical trials, data collection should be continued beyond the date of disease progression, to include the date of initiation of the next line of anti-CTCL therapy.

DOCB = duration of clinical benefit; nbUVB/PUVA = narrowband UVB phototherapy and psoralen-UVA photochemotherapy; TSE = total skin electron therapy.
